# Delayed diagnosis of Brugada syndrome in a patient with aborted sudden cardiac death and initial negative flecainide challenge

**DOI:** 10.1002/ccr3.1198

**Published:** 2017-10-31

**Authors:** Samuel Chauveau, Olivier Le Vavasseur, Philippe Chevalier

**Affiliations:** ^1^ Department of Rhythmology Hospices Civils de Lyon Louis Pradel Cardiovascular Hospital Lyon France; ^2^ Department of Cardiology Northwest Hospital Gleizé France; ^3^ Lyon Reference Center for inherited Arrhythmias Louis Pradel Cardiovascular Hospital Lyon France

**Keywords:** Arrhythmia, brugada syndrome, electrocardiogram, flecainide

## Abstract

A negative flecainide challenge does not rule out Brugada syndrome even in the presence of nonfatal cardiac arrest as the first manifestation of the disease. This should prompt clinicians to ensure long‐term ECG follow‐up and consider repeating a drug test with another sodium channel blocker.

A 29‐year‐old man was admitted for out‐of‐hospital cardiac arrest related to ventricular fibrillation (VF) that was successfully terminated after cardiopulmonary resuscitation and one external biphasic shock at 200 J. Ventricular fibrillation occurred while the patient was having lunch. The patient had no past medical record and no familial history of sudden cardiac death.

On admission, ECG revealed sinus rhythm with incomplete right bundle branch block and no evidence of cardiac ischemia or channelopathy (Fig. 1, panel A). Coronary angiography and transthoracic echocardiography were normal. Flecainide challenge (1.6 mg/kg, i.e. 150 mg over 10 min) did not unmask Brugada pattern (Fig. 1, panel B), and isoproterenol provocation testing (45 μg/min over 3 min) did not induce ventricular arrhythmias. Treadmill testing was unremarkable. Given resuscitated cardiac arrest with documented VF, a subcutaneous implantable cardioverter defibrillator (EMBLEM™ S‐ICD) was implanted. At 4‐month follow‐up visit, ECG revealed typical Brugada pattern (Fig. 2, panel A) and 2 months later, the patient experienced VF recurrence during nighttime that was successfully converted to sinus rhythm after one internal shock at 80 J (Fig. 2, panel B). Hydroxyquinidine was started at a daily dose of 300 mg.

**Figure 1:**
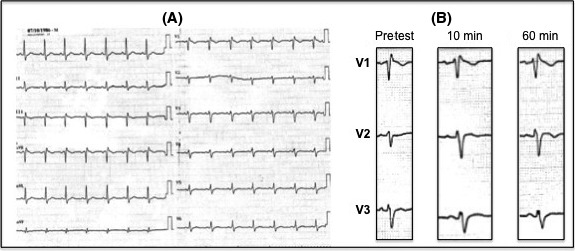
**Panel A**. ECG on admission revealed incomplete right bundle branch block. **Panel B.** Repeated ECG before (pre‐test), during (at the end of the 10‐minute infusion) and after (50 minutes after the end of the infusion) intravenous flecainide administration (150 mg over 10 minutes) showed no significant changes in ST segment especially in right precordial leads.

**Figure 2:**
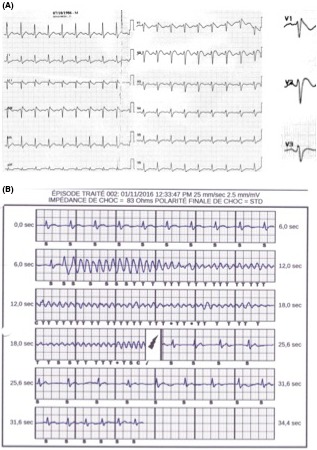
**Panel A.** Twelve‐lead ECG recorded 4 months after the patient experienced cardiac arrest demonstrated typical Brugada ECG pattern with coved ST‐segment elevation with type 1 morphology ≥2 mm in leads V1 and V2. **Panel B**. Stored electrograms from the subcutaneous implantable cardioverter defibrillator showed a spontaneous episode of ventricular fibrillation successfully converted to sinus rhythm after one internal shock at 80 J (thunderbolt).

This case evidences that a negative flecainide challenge does not rule out Brugada syndrome even in the presence of aborted cardiac arrest as the first manifestation of the disease. This is of crucial importance as recognition of Brugada syndrome should lead to familial screening and recommendations concerning lifestyle changes.^1^ Reported incidence of negative response to flecainide challenge in patients with definite Brugada syndrome varies between 14.5 and 32 percent of which approximately 50 percent are symptomatic.^2^, ^3^ However in these studies, syncope but not aborted cardiac arrest was the clinical manifestation of the disease. Moore et al.^4^ reported a possible late diagnosis of Brugada syndrome in a patient with previous cardiac arrest and a negative flecainide challenge, but electrical changes were documented 9 years after the event and were based on electrograms recorded before and briefly after defibrillation testing. When compared to flecainide, ajmaline has been reported to be more effective in unmasking Brugada pattern on the ECG and this difference has been linked to less ajmaline‐induced I_to_ inhibition.^2^ Yet, higher diagnostic value of ajmaline challenge compared to flecainide remains uncertain as it has not been confirmed in larger population studies.^5^ We did not perform ajmaline challenge on initial evaluation, but ECG at 4‐month follow‐up and recurrent VF unambiguously demonstrated a malignant form of Brugada syndrome. The main limitation of this case report lies in the absence of ECG recordings with the right precordial leads placed at the 2nd or 3rd intercostal spaces either at baseline or during the flecainide challenge. Indeed positioning the right precordial leads upwardly can unmask Brugada ECG pattern. However, as type I Brugada ECG pattern became evident at 4‐month follow‐up visit while the right precordial leads were placed at the 4th intercostal space, we believe the absence of Brugada ECG pattern during the flecainide challenge resulted from the imperfect sensitivity of the flecainide challenge rather than from the positioning of the electrodes.

In conclusion, a negative flecainide challenge in a patient experiencing aborted sudden cardiac death is not sufficient to exclude Brugada syndrome and should prompt clinicians to ensure long‐term follow‐up with repeated ECG.

## Authorship

SC: wrote the manuscript and the revisions. OLV: acquired the data. PC: wrote the manuscript.

## Conflict of interest

None declared.

## References

[1] Priori, S. G. , A. A. Wilde , M. Horie , Y. Cho , E. R. Behr , C. Berul , et al.2010 HRS/EHRA/APHRS expert consensus statement on the diagnosis and management of patients with inherited primary arrhythmia syndromes: document endorsed by HRS, EHRA, and APHRS in May 2013 and by ACCF, AHA, PACES, and AEPC in June 2013. Heart Rhythm: the Official Journal of the Heart Rhythm Society 10:1932–1963. 10.1016/j.hrthm.2013.05.014 24011539

[2] Wolpert, C. , C. Echternach , C. Veltmann , C. Antzelevitch , G. P. Thomas , S. Spehl , et al. 2005 Intravenous drug challenge using flecainide and ajmaline in patients with Brugada syndrome. Heart Rhythm: the Official Journal of the Heart Rhythm Society 2:254–260. 10.1016/j.hrthm.2004.11.025 PMC1474213 15851314

[3] Priori, S. G. , C. Napolitano , M. Gasparini , C. Pappone , P. Della Bella , M. Brignole , et al. 2000 Clinical and genetic heterogeneity of right bundle branch block and ST‐segment elevation syndrome: A prospective evaluation of 52 families. Circulation 102:2509–2515. 11076825 10.1161/01.cir.102.20.2509

[4] Moore, P. T. , and G. C. Kaye . 2015 Possible late diagnosis of the Brugada syndrome in a patient presenting with a primary cardiac arrest. Europace 17:1839. 25911350 10.1093/europace/euv089

[5] Probst, V. , J. B. Gourraud , S. Chatel , J. Mansourati , F. Sacher , D. Babuty , et al. 2013 Comparison between flecainide and ajmaline challenge in Brugada syndrome patients. European Heart Journal 34(suppl 1):P2295.

